# Editorial: Fetal analgesia: a conundrum for the physiologist, a tenet for the surgeon

**DOI:** 10.3389/fpain.2024.1478396

**Published:** 2024-09-02

**Authors:** Carlo V. Bellieni, Kim K. Doheny, Maria A. Flores Munoz, Gloria Pelizzo

**Affiliations:** ^1^ Department of Neonatology, University of Siena, Siena, Italy; ^2^ Department of Neural and Behavioral Sciences, College of Medicine, The Pennsylvania State University Hershey, Hershey, PA, United States; ^3^ General Hospital of Mexico, Mexico City, Mexico; ^4^ Department of Biomedical and Clinical Science, University of Milan, Buzzi Children's Hospital, Milan, Italy

**Keywords:** pain, analgesia, fetal pain, pregnancy, fetus

**Editorial on the Research Topic**
Fetal analgesia: a conundrum for the physiologist, a tenet for the surgeon

**Figure 1:**
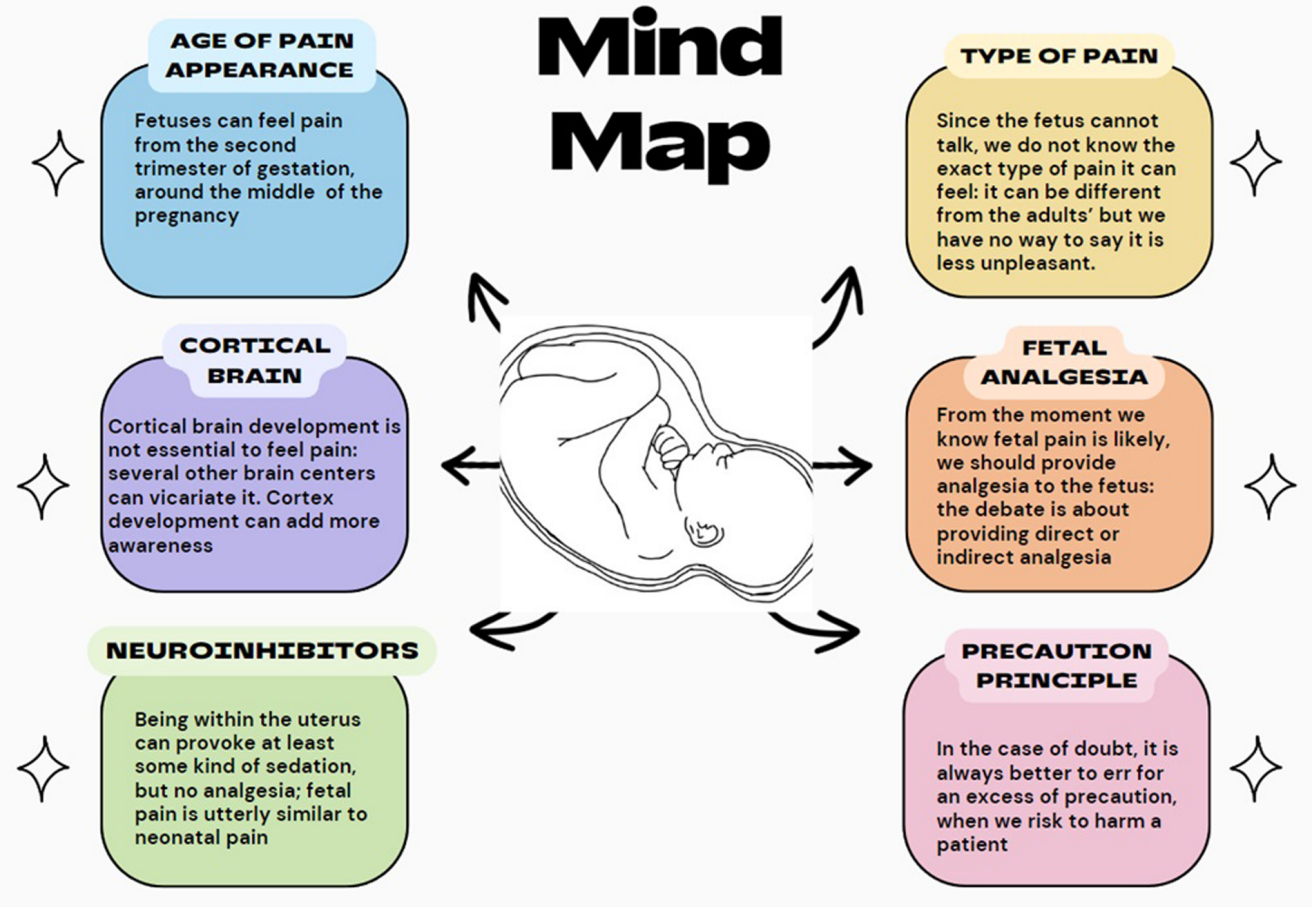
Main evidence about fetal pain.

Neonatal pain has become commonly accepted by all, while fetal pain has been less well described and often not accepted. At the end of the last century, it was assumed that newborns did not feel pain, and they underwent surgery without the necessary analgesia (1). Fetal pain is the other side of the newborn's pain because the fetus is exactly a newborn who has not yet left the womb. The hypothesis that inside the uterus there is a special protection against pain has been refuted, so much so that even the Royal College of Obstetricians and Gynecologysts in its 2022 document (2) no longer talks about it. Once, saying that the fetus could feel pain was just to say “the emperor is naked!” (3) But this special issue on fetal pain sets a milestone. With tact and competence, it describes the evidence of fetal pain. Now it remains to highlight what type of pain it is, given that the development of the perception and of the self-consciousness is still at its early stages; and from what gestational age fetal pain is a real fact (Figure 1). The challenge of this special issue consists in not wanting to draw utter conclusions on these questions. It just deals with fetal pain, observes the circumstances of this pain, describes its existence in the second trimester; then it leaves room for future research. This is a sign of cultural honesty by the authors, in a field where there is a tendency to deny fetal pain or to exaggerate its presence for political reasons.

Fetal pain is really a challenge. Today we can perform fetal surgery, and it would be truly atrocious not to use anesthetics if the fetus felt pain; when the fetus cannot feel pain anesthesia is useless; when they can it is mandatory: we need to understand from what age we should use fetal anesthetics in case of fetal surgery.

Fetal direct interventions are becoming increasingly safer, as shown by the beautiful series of cases of *in utero* transfusions shown by Lanna et al. now we can really treat the fetus like any anemic patient. Rather, it is a patient who experiences various types of stress, including painful stress, as Calcaterra et al. magistrally explain: from mid-gestation, and may be from the beginning of the 2nd trimester, both the thalamus and the limbic system are active and can perceive pain as Thill review shows. Unfortunately, Duci et al. show us that there are still no universally recognized guidelines for the treatment of fetal surgical pain. And Canepa et al. tell us that many prejudices still prevent recognizing a definition of the word and the experience of pain that applies to all human beings, from those who are non-verbal because they are too small or still unborn or because they are in a coma or because have mental disabilities.

This discussion underlines the subtitle of this special issue: “a tenet for the surgeon and a conundrum for the physiologist.” Because surgeons understand they should “not harm,” and the risk of provoking pain is well evident in the case of the fetus; but physiologists should challenge the too easy assertions that describe fetal pain simply with a *yes* or a *not*.

We have attained the evidence of fetal pain. Now we should solve three mysteries correlated with it.

We are not sure when this ability to perceive begins. It starts in the weeks around the middle of pregnancy, in a range around the 20th week of gestation, but the exact starting day is still a mystery.

We also acknowledge our inability to understand the type of pain that the fetus perceives, given that it cannot express it in words. We know which are the structures necessary and unnecessary to feel pain, and we know when they start firing; few studies are done directly measuring the production of stress hormones during fetal pain and they can help, but they are not enough: thus, we do not know if fetal pain is a burning, dry, penetrating, superficial pain, and to what level of consciousness it reaches. This is another mystery.

We can say that fetal pain is a phenomenon that encompasses fetal consciousness, and this is important. Because the fetus has its own consciousness (4). When we talk about consciousness, we mean the sense of pleasant and unpleasant sensations, which is distinct than awareness: the former depends on deep areas of the brain, the latter on the cortex and the experiences of one's body. We know that the fetus has a form of consciousness (it will clearly remember the sensations experienced before being born), but it does not have awareness, for which specific cortical centers are needed. So, we wonder how an undoubtably unpleasant event is specifically “unpleasant” for the fetus. Another mystery, isn't it?

This issue is an appeal to all researchers to gather their efforts to share light on these mysteries. It is a simple but effective opportunity to know this problem, acknowledging its limits, and go forward in searching the truth.
